# Rapid expansion of human impact on natural land in South America since 1985

**DOI:** 10.1126/sciadv.abg1620

**Published:** 2021-04-02

**Authors:** Viviana Zalles, Matthew C. Hansen, Peter V. Potapov, Diana Parker, Stephen V. Stehman, Amy H. Pickens, Leandro Leal Parente, Laerte G. Ferreira, Xiao-Peng Song, Andres Hernandez-Serna, Indrani Kommareddy

**Affiliations:** 1 Department of Geographical Sciences, University of Maryland, College Park, MD, USA.; 2 College of Environmental Science and Forestry, State University of New York, Syracuse, NY, USA.; 3 Image Processing and GIS Lab (LAPIG), Federal University of Goiás (UFG), Goiânia, Brazil.; 4 Department of Geosciences, Texas Tech University, Lubbock, TX, USA.

## Abstract

Across South America, the expansion of commodity land uses has underpinned substantial economic development at the expense of natural land cover and associated ecosystem services. Here, we show that such human impact on the continent’s land surface, specifically land use conversion and natural land cover modification, expanded by 268 million hectares (Mha), or 60%, from 1985 to 2018. By 2018, 713 Mha, or 40%, of the South American landmass was impacted by human activity. Since 1985, the area of natural tree cover decreased by 16%, and pasture, cropland, and plantation land uses increased by 23, 160, and 288%, respectively. A substantial area of disturbed natural land cover, totaling 55 Mha, had no discernable land use, representing land that is degraded in terms of ecosystem function but not economically productive. These results illustrate the extent of ongoing human appropriation of natural ecosystems in South America, which intensifies threats to ecosystem-scale functions.

## Teaser

A comprehensive record of the land use history of South America reveals that human impact on land increased by 60% since 1985.

## INTRODUCTION

Our improved ability to monitor changes on the Earth’s surface via time series of satellite-based Earth observations is timely, given increasing rates of human-induced environmental change (*1*–*3*). Growing global populations, increased levels of development, and the resulting greater interconnectedness have led to heightened demand for goods such as food, timber, minerals, and fuels, the production of which requires the transformation and appropriation of natural ecosystems through land cover and land use change (*4*). Land cover/land use change is the dominant factor altering the global land surface (*3*) and the most significant of anthropogenic impacts on the environment (*5*), with implications for ecosystem functioning, biogeochemical cycles, and biophysical processes, among others. The degradation of natural land covers also has important consequences, albeit less well-quantified (*6*).

Precise information on the expansion of commodity land uses across the globe does not currently exist. We can use commodity production data from the Food and Agriculture Organization (FAO) as evidence of the regions in the world that have experienced the most commodity land use growth, with the caveat that production rates can vary significantly across geographies. Figure S1 shows that since 1985, South America has been the site of the greatest increases in the production of soy, sugarcane, and nonconiferous pulpwood. Most notably, South America went from producing a quarter of the world’s growing soy supply in 1985 to more than half by 2018. South America also produces the largest share of the world’s beef supply and is only second to eastern Asia in terms of increases in corn production since the start of our study period. Altogether, commodity production data indicate that, as North America’s share of global agricultural commodity production fell, South America played a leading role in the overall increase of agricultural commodity production (fig. S1). As increases in agricultural commodity production are linked to commodity land uses, no region on Earth is likely to have experienced the scale of land conversion for the sake of agricultural commodity production that South America has. At the same time, South America is home to some of the world’s most important ecosystems, such as the Amazon, Cerrado, and Chiquitania forests. The Amazon is one of the most undisturbed regions remaining on Earth (*7*), constitutes a massive carbon sink (*8*), regulates critical hydrologic and climate systems (*9*), and provides habitat for uncounted numbers of species (*10*). The Cerrado savanna is also of critical importance as it is a global biodiversity hotspot (*11*). The Chaco and Chiquitania ecosystems make up an important continuous extent of natural vegetation, one of the largest remaining in South America (*12*). Tensions between expanding the area used for the production of agricultural commodities and preserving important natural ecosystems have long existed and have led to efforts to map and quantify changes in land cover and land use to better understand the dynamics, drivers, and consequences of change (*4*).

Various initiatives focus on estimating the extent of intact or wild ecosystems (*13*–*15*), often using models based on an aggregation of disparate map products. Here, we improve on those methods using an internally consistent analysis of more than 30 years of time series satellite data to estimate human impact on natural land in South America, ranging from forest degradation to land use conversion, and including transitions between land uses. We first created maps of natural land cover, cropland, tree regrowth, and tree plantations using Landsat earth observation imagery. We then used these maps to select a stratified random sample of 1000 30-m pixels and labeled the land cover/land use class of each sample pixel for every year from 1985 to 2018. Area estimates of major land change dynamics across South America along with associated uncertainty measures were derived from the reference sample data, adhering to current international reporting guidelines (*16*–*18*). We examine these land change dynamics at continental, national, and ecozonal scales.

## RESULTS

### Human impact on natural land

**Fig. 1:**
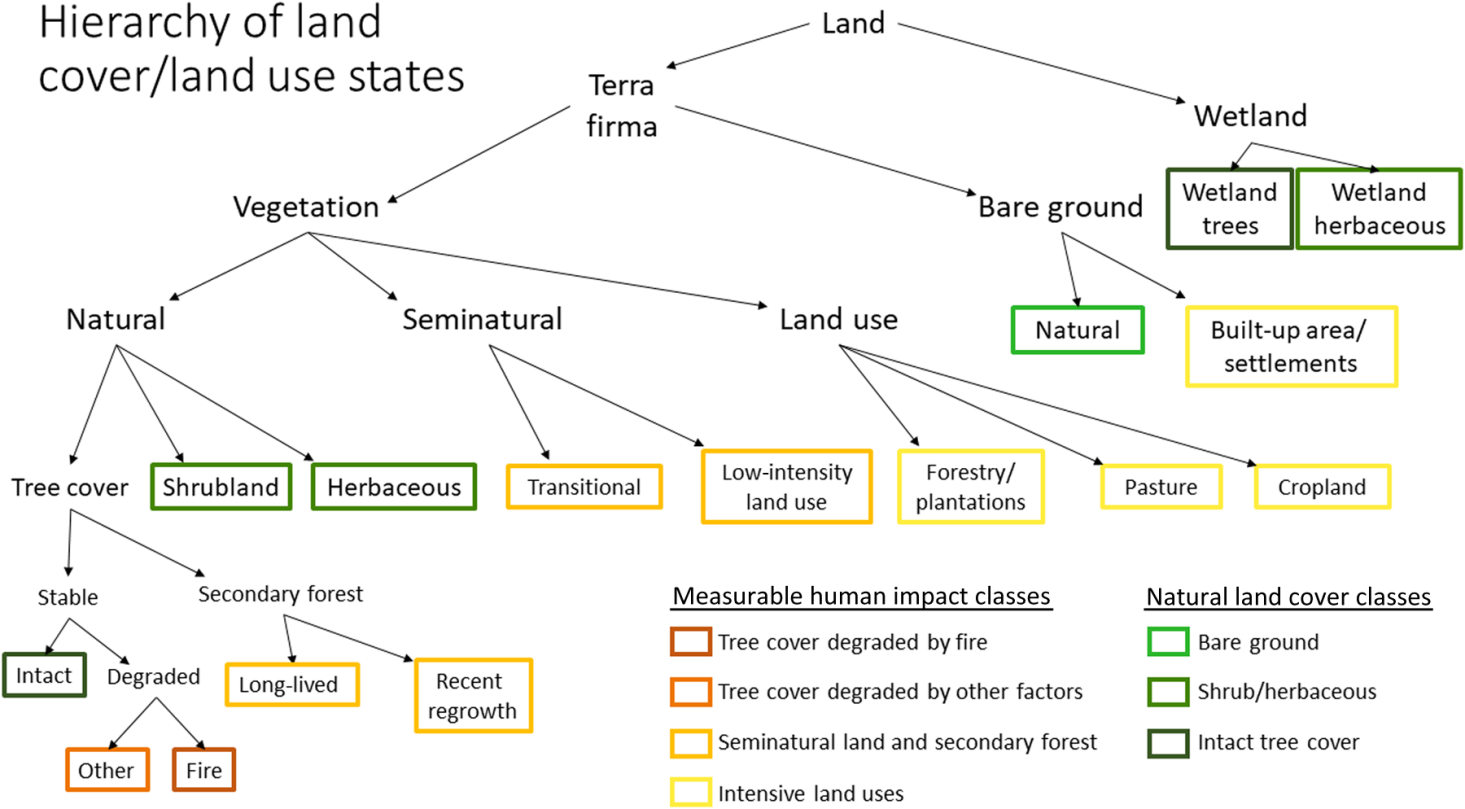
Hierarchical legend used for estimating human impact on natural lands. Intensive land uses include built-up area, settlements, cropland, pasture, and forestry and tree plantations.

**Fig. 2:**
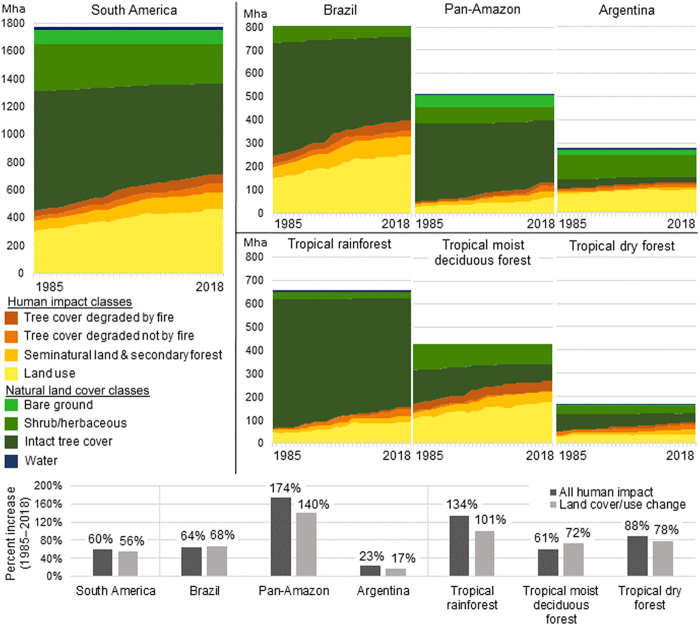
Human impact across South America. Human impact across South American regions categorized by degree of modification or conversion and the intensity of land use and natural land cover type. “Pan-Amazon” includes the combined area of Colombia, Venezuela, Peru, Bolivia, Ecuador, Suriname, French Guiana, and Guyana and excludes Brazil. Lower insert shows percent increase over the study period for all human impact classes and for land cover/use change classes only (“seminatural land and secondary forest” and “land use” categories, excluding tree cover degradation dynamics).

We define human impact on natural land cover as the combined area of land use, seminatural land cover, degraded natural tree cover, and secondary forest, as can be observed at the Landsat spatial resolution (see hierarchy of land cover/land use; Fig. 1). Human impact on natural land cover in South America increased by 60% since 1985, reaching 713.7 ± 32.3 Mha, or 40% of the continent’s landmass, by 2018 (the uncertainty is expressed as ±1 SE of the estimate) (Fig. 2). This outcome corresponds to a 20% decrease in unaltered natural land cover in a 34-year time period. The area of land under intensive land uses (cropland, pasture, tree plantations, and built-up area) increased by 56%, totaling 465.7 ± 25.9 Mha in 2018. The amount of land under low-intensity, small-scale, abandoned, or ephemeral land uses (collectively referred to as seminatural land) increased by 66%, totaling 114.5 ± 16.3 Mha in 2018. Altogether, 17% of 2018 tree cover extent in South America was impacted by one or more degradation events at some point since 1985.

Human impact on natural land increased more in Brazil than in any other country (64% increase, totaling 398.8 ± 19.7 Mha by 2018). The largest relative increase in human impact occurred in the broad region we refer to as the Pan-Amazon excluding Brazil (Colombia, Venezuela, Peru, Bolivia, Ecuador, Suriname, French Guiana, and Guyana combined), where human impact increased by 174%, reaching 131.6 ± 17.8 Mha in 2018. Argentina, conversely, had much lower rates of new human appropriation of natural land cover: The human impact on natural land grew by a comparatively moderate 23%, reaching 130.0 ± 14.7 Mha in 2018.

The tropical rainforest ecozone, which is primarily made up of the Amazon rainforest, experienced an increase in human impact on natural land of 134%, increasing to 155.5 ± 19.2 Mha in 2018. In this ecozone, 8% of the remaining natural tree cover was degraded by 2018, with a majority of initial degradation (77%) due to factors other than fire, such as logging. In the tropical moist deciduous forest ecozone, which consists mainly of the Cerrado and humid Chaco biomes, human impact increased by 61%, much of it due to increases in intensive land use. Unlike the tropical rainforest ecozone, the vast majority of tree cover degradation in tropical moist deciduous forests was due to fire, which accounted for 87% of degraded tree cover. Human impact in the tropical dry forest ecozone (Caatinga and dry Chaco biomes) increased by 88%, totaling 89.5 ± 11.8 Mha in 2018. By 2018, both the tropical moist deciduous forest ecozone and the tropical dry forest ecozone had more land impacted by human activity than intact natural land, a reversal from 1985.

### Land cover/land use change, 1985 to 2018

#### Continental-scale trends

**Fig. 3:**
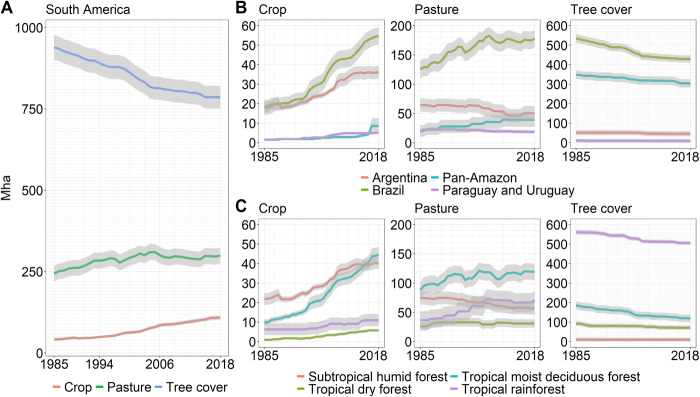
Land cover/land use trends across South America. Land cover/land use trends of cropland, pasture, and tree cover area for (**A**) the continent, (**B**) administrative regions, and (**C**) ecozones. The shaded area corresponds to 1 SE of the estimate. Note that the upper bounds of the charts for crop, pasture, and tree cover vary. All vertical axes are in million hectares.

**Fig. 4:**
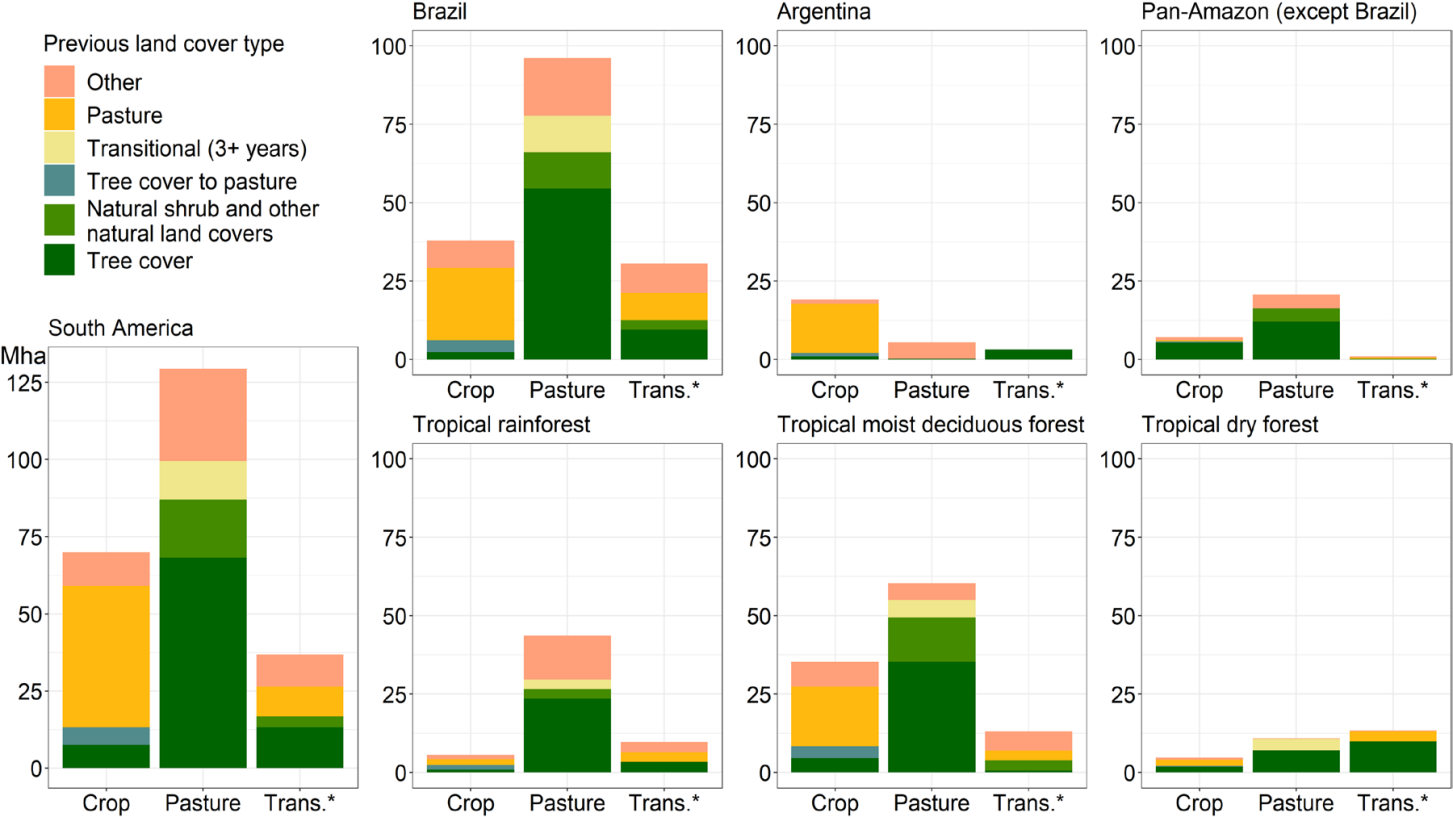
Land cover/land use transitions across South America. Area of new cropland, pasture, and transitional land (>3 years) per region. Colors represent previous land cover type. The areas shown here correspond only to areas having experienced a single (one-step) transition over the study period, with the exception of the “tree cover to pasture to crop” category. Land that underwent more than one land cover/land use change event over the study period is included in the “other” category. All vertical axes are in million hectares.

Nearly one-fifth of the South American continent underwent some type of land cover/land use change over our study period. The single most significant land change dynamic was the conversion of intact and degraded natural tree cover (hereafter referred to as tree cover for simplicity; refer to Fig. 1 for land cover/land use hierarchy), which totaled 153.6 ± 16.8 Mha and represented half of all continental-level change (Fig. 3A). Tree cover decreased steadily at an average rate of 6.4 Mha/year from 1985 to 2004, at which point the rate of loss slowed considerably to an average of 2.3 Mha/year (Fig. 3A). This continental-level trend was largely driven by a marked decline in tree cover loss in Brazil after 2004, which largely persisted through 2018. The reduction was likely due to the enforcement of land use regulations and to increased scrutiny and action on improving supply chains and the granting of credit, all of which led to a decrease in new deforestation (*19*). By 2018, 16% of South America’s 1985 tree cover area was lost. A significant portion of this tree cover (44%) was converted to pasture by 2018 via a one-step land cover/use change transition (Fig. 4). The second most common 2018 fate of the 1985 tree cover (9%) was transitional land, followed by tree cover converted directly (within 3 years) to cropland (5%). Transitional land corresponds to land where a change in cover due to human intervention is observable but with no signs of an established subsequent land use (see table S2). The estimated area of land that underwent a two-step tree cover to pasture to crop transition during the study period was 5.6 ± 1.2 Mha (Fig. 3), representing 4% of all tree cover loss.

The second largest category of change consisted of gross pasture gain, which totaled 129.4 ± 16.7 Mha. Gross pasture loss was also a major change dynamic, totaling 74.2 ± 7.9 Mha. From 1985 to 2004, total pasture area increased at an average rate of 3.5 Mha/year, after which pasture area stabilized at around 300 Mha, netting a 23% increase over the study period (Fig. 3A). The stabilization of continental-level pasture area is not due to a lack of land use change but rather to the fact that regional losses and gains partially compensate each other: Brazil and the Pan-Amazon region’s gains are compensated by losses in Argentina, Paraguay, and Uruguay (Fig. 3B).

Other major dynamics of land change include increases in the area of cropland, tree plantations, short tree crops, and transitional land. Cropland area grew consistently across the continent at an average rate of 2.0 Mha/year, resulting in an increase in area by a factor of 2.6 over the study period (Fig. 3A). The majority of new cropland area (66%) was created through the conversion of pastures (Fig. 4). The combined area of tree plantations and short tree crops nearly quadrupled since 1985, reaching 25.6 ± 4.6 Mha by 2018 (288% net increase). Natural tree regrowth observed during the study period totaled 37.8 ± 9.6 Mha. By 2018, there were 55.4 ± 12.7 Mha of long-lasting (>3 years) transitional land across the continent, which resulted from the conversion of tree cover (36%) and other natural land covers (10%) as well as from the abandonment of pastureland (26%) (Fig. 4).

#### National-scale trends

Brazil, the largest country in South America, exhibited the most extensive land use change. The area of natural tree cover in 2018 was 429.13 ± 18.6 Mha, a 20% decrease in extent since 1985 (Fig. 3). The rate of tree cover loss averaged 4.8 Mha/year until 2004, when it slowed to an average of 1.0 Mha/year. Of the total tree cover loss area in Brazil, 52% was converted to pasture (Fig. 3). Pasture area increased by 42% (net change) and experienced an inflection point in 2004 when the rate of increase slowed from 2.9 to 0.2 Mha/year (Fig. 3B). An important factor in the leveling of pasture expansion is intensification, including cultivated pastures, semi-containment, and other practices (*20*, *21*). Cropland area in Brazil nearly tripled since 1985 (Fig. 3B), and the combined area of tree plantations and short tree crops increased by a factor of 3.4. New cropland area in Brazil was sourced primarily (61%) from converted pastures (Fig. 4). Compared to Brazil, Argentina had lower levels of change. The most significant changes in Argentina were a doubling of cropland area and a 22% net decrease in pasture area (Fig. 3). These two changes were connected: 80% of pastureland lost over the study period was converted to cropland (Fig. 4). Pasture area in Argentina was stable at around 64 Mha until 2003, after which it started to decrease (Fig. 3B).

#### Ecozonal-scale trends

Tropical rainforests lost 55.4 ± 8.5 Mha of tree cover, representing a 10% decrease from 1985 levels, and the tropical most deciduous forest lost 66.4 ± 11.3 Mha, representing 36% of the 1985 tree cover extent. The rate of tree cover loss slowed markedly after 2004 in both these ecozones. Tree cover was primarily converted to pasture in both ecozones, with 23.6 ± 7.2 Mha of land being converted from tree cover to pasture in the tropical rainforest, and 35.3 ± 8.6 Mha in the tropical moist deciduous forest. Consequently, pasture area increase was substantial in both ecozones: In the tropical rainforest ecozone, pasture area increased steadily until 2004, after which it stabilized at around 70 Mha (91% net increase). Loss of pasture area in the subtropical humid forest ecozone compensated for gains in other ecozones, resulting in stable pasture area at the continent level after 2004.

Tropical moist deciduous forests were the epicenter of cropland expansion in South America as crop area in this ecozone nearly quintupled during the study period, totaling 44.6 ± 4.2 Mha by 2018 (Fig. 3C). New cropland was mainly created through pasture conversion (54%) but also through the direct conversion of tree cover (13%) and the conversion of pasture that had previously been tree cover (11%). Cropland area also increased significantly in the subtropical humid forest ecozone (19.8 ± 1.4 Mha of gross cropland gain), netting an increase of 84% over its 1985 extent (Fig. 3C). New cropland in this ecozone came about almost exclusively through the conversion of pastures (92%) (Fig. 4).

## DISCUSSION

Humans have markedly altered environments through the conversion of natural vegetation pasture, crop, and other land uses, as well as the degradation of natural vegetation through logging, fire, and other disturbance types. The appropriation of natural vegetation is largely driven by the increased demand of agricultural commodities worldwide. Global agricultural commodity data (fig. S1) point to the fact that the South American continent has likely been the site of the greatest expansion of commodity land uses in the last decades, and the results presented here quantify the associated land change dynamics. In South America, the increase in human impact on natural land cover over the past 34 years has averaged 8.1 Mha/year, which is equivalent to 21.6 soccer fields/min.

Changes in land cover have important consequences to climate at regional and global scales by altering fluxes of energy, water, and greenhouse gas emissions (*22*–*24*). For example, deforestation in the Amazon basin has been shown to increase near-surface air temperature and decrease evapotranspiration, and after a certain threshold, these temperature and precipitation changes could cause a shift in vegetation to a Cerrado-like ecosystem (*9*, *25*). In addition, land cover/land use change has important impacts on biodiversity. Land change is expected to be the single largest driver of global biodiversity loss by 2100 because of its limiting effects on habitat availability and consequent species extinctions (*26*). Because the tropical and temperate forests of South America are projected to have the highest rates of land use change, biodiversity losses are expected to be most acute in South America (*26*). Species extinction alters ecosystem productivity and decomposition at rates comparable to those of climate warming (*27*) and has important consequences to the invasibilty, stability, and resilience of ecosystems (*28*). The climatic, hydrologic, nutrient cycling, and biodiversity impacts of land cover/land use change jeopardize ecosystems’ functioning and their ability to provide the ecosystem services on which long-term human well-being is dependent. Food production, access to fresh water, access to clean air, and protection from extreme climatic events such as droughts and floods are only some of the myriad of goods and services provided by well-functioning ecosystems. Considering that extensive lands remain suitable for further expansion of commodity land uses (*29*) and that recent expansion has occurred even in low-suitability areas (*30*–*33*), we may expect further loss of natural land cover, threatening the maintenance of ecosystem services for major biomes such as the Amazon, Cerrado, Chaco, and Chiquitania (*34*–*38*). Given these threats, results here are a clarion call to improved land use policy formulation, implementation, and enforcement.

In South America, the most extensive human impact on land was the conversion of natural vegetation into pastureland. Beef production in South America has historically been characterized by its extensive rather than intensive nature (*21*, *39*). It has been estimated that the productivity of pastures in Brazil is less than half of their carrying capacity (*40*), despite recent significant gains in productivity (*21*, *39*). Low-intensity, low-productivity pastureland replacing natural vegetation and the widespread phenomenon of cropland replacing pastureland are two important dynamics that reflect the overall intensification of land use across South America. Given the tripling of cropland area over the study period and the likely continued expansion of the commodity crop footprint (*41*, *42*), an understanding of the interplay of crop and pastureland is needed (*43*). While policies such as the soy moratorium in Brazil have proven to be nominally effective (*19*, *44*, *45*), leakage effects and the displacement of other land uses such as pastures into deforestation frontiers illustrate the need for more comprehensive monitoring (*46*). Our estimate of annual tree cover loss in Brazil since 2004, the year of minimum Amazon deforestation (*47*), averaged more than 1 Mha/year, with 70% being converted to pasture, suggesting a sizeable loophole for narrowly tailored policies. Further evidence of the trend of land use intensification across the continent is the increase in economic output per hectare of land under agricultural production (see Materials and Methods and fig. S2). In Brazil and Argentina, for example, the value added from the agricultural sector has increased more quickly than the area of cropland in each of these countries (fig. S2A), which points to an increase in yield. Increases in yield can be attributed to more intensive land use management through the use of biotechnology, agricultural inputs, and improved agricultural practices (mechanization, irrigation, double-cropping, etc.) (*48*).

Beyond intensive land uses, 55.4 ± 12.7 Mha of land (3% of the South American continent’s landmass) were converted from a natural state but were not used for any discernable economic purpose. This long-lasting transitional land category may be associated with land speculation or land-tenure establishment (*49*, *50*). It should be of particular concern to policy-makers and other stakeholders, as it represents land area that has been compromised in terms of the provisioning of ecosystem services yet is relatively unproductive in terms of economic output. Monitoring natural land cover from initial disturbance to possible conversion is necessary to fully understand land use pathways, including establishment of tenure, land banking, and eventual production of high-value commodities.

## MATERIALS AND METHODS

### Landsat data processing

All Landsat TM (Thematic Mapper), ETM+ (Enhanced Thematic Mapper Plus), and OLI/TIRS (Operational Land Imager/Thermal Infrared Sensor) observations from 1 January 1985 to 31 December 2018 were downloaded and processed using the methodology described by Potapov *et al.* (*51*, *52*). Processing steps included conversion to at-sensor radiance, per-pixel quality assessment, reflectance normalization, and aggregation into 16-day composites. The 16-day composites were then used as inputs to time series metrics for annual and multiyear mapping tasks. Metrics are statistical derivatives of time series imagery that represent a generalized feature space appropriate for large area mapping (*53*–*55*).

### Multi-epochal metrics

**Fig. 5:**
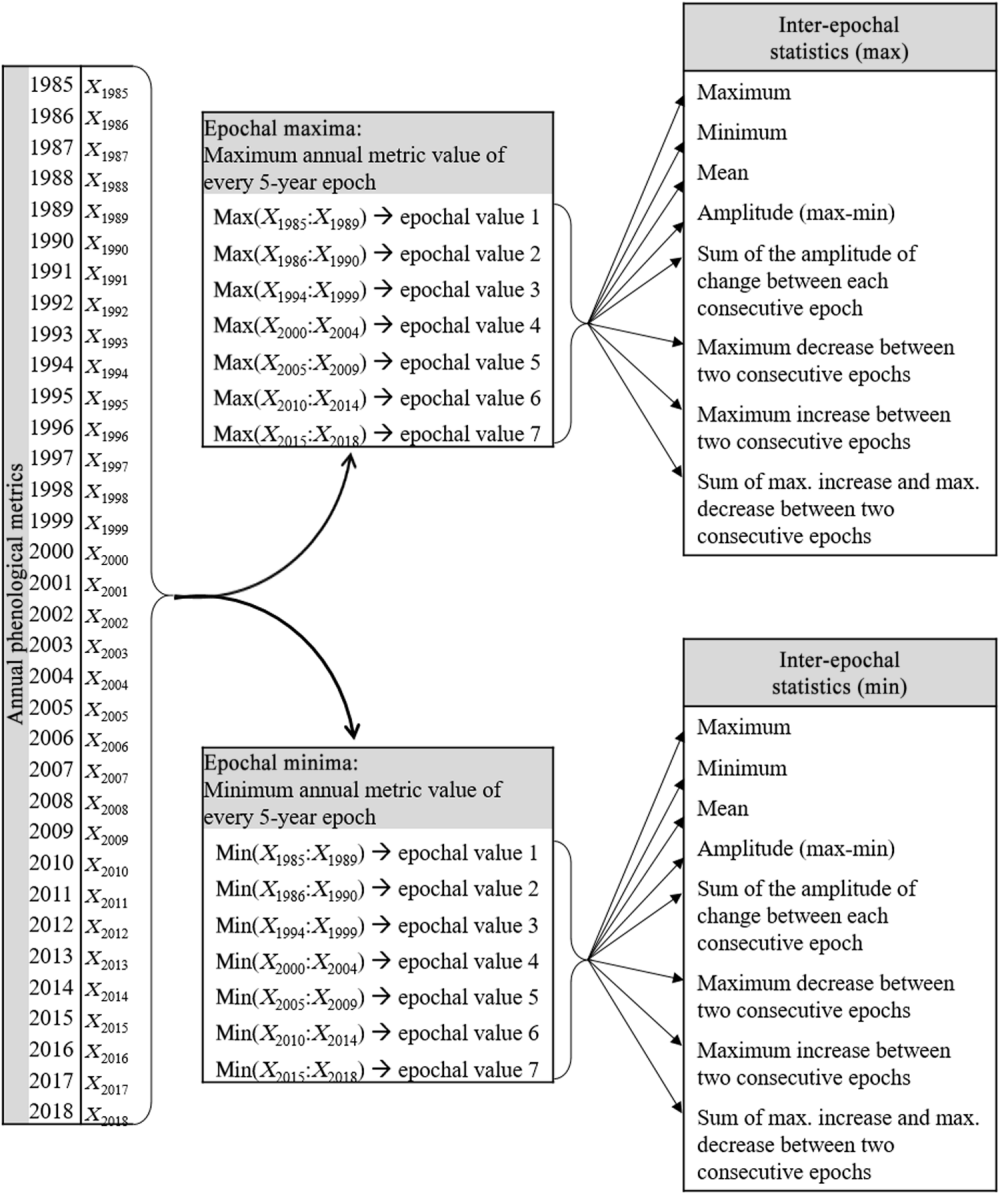
Schematic representation of the multi-epochal metric set. Annual phenological metrics used include the penultimate highest value, penultimate lowest value, and interquartile mean of annual red, NIR, SWIR 1 (1.6 μm), SWIR 2 (2.2 μm), and NDVI. *X_i_* represents the value of a given annual phenological metric for year *i*. The inter-epochal metric set is composed of all “max” and “min” inter-epochal statistics for each of the 15 selected annual phenological metrics, totaling 240 new metrics encompassing 34 years of spectral reflectance.

An additional metric set was created using the annual phenological metrics described above as inputs. Instead of using annual change detection metrics, we created this new set of metrics to capture land cover change across the entire 34-year time period, regardless of timing. To create this multi-epochal metric set, we selected a set of metrics from the annual metric set: the second highest, second lowest, and mean of the 25th to 75th percentile of observations of the red, near infrared (NIR), shortwave infrared (SWIR) 1 (1.6 μm), SWIR 2 (2.2 μm), and NDVI (normalized difference vegetation index). We then aggregated each of these into 5-year epochs (six 5-year epochs and one 4-year epoch corresponding to 2015 to 2018). Epochs were used to capture land changes over relatively short time frames and to normalize the 34-year input data, which vary markedly in data richness over the study period. For each metric of each band, we retained the maximum and the minimum value of each epoch. At this stage, for each of the selected annual metrics, we had seven values corresponding to the 5-year maxima of each epoch and seven values corresponding to the 5-year minima of each epoch. From these seven values, we extracted eight new statistics: (i) maximum value, (ii) minimum value, (iii) mean value, (iv) amplitude, (v) sum of the absolute amplitude of change between each consecutive epoch, (vi) maximum decrease between two consecutive epochs, (vii) maximum increase between two consecutive epochs, and (viii) sum of maximum increase and maximum decrease between two consecutive epochs (Fig. 5).

### Annual cropland mapping

Annual multitemporal phenological metrics were generated from 16-day inputs to create a consistent input for mapping annual cropland extent [refer to (*51*) for a complete description of these metrics]. Yearly maps of cropland, which we define as areas of intensive row crop agriculture, were created for the entire South American continent. To achieve this, we first performed a supervised classification of cropland using a bagged classification tree algorithm (*56*, *57*) and manually labeled Landsat training data corresponding to 2016, 2017, and 2018. Using training data and corresponding metrics for three different years allowed us to create a more stable classification tree model that could separate crops from other land covers despite annual variation. We then applied this turn-key model to every yearly metric set going back to 1985 to obtain yearly cropland maps.

### Stable land cover, tree plantation, and natural tree regrowth mapping

Yearly metrics are not appropriate for mapping natural tree regrowth and tree plantations because mapping a gradual process such as the growth of trees requires longer time series. The multi-epochal metric set captures changes in surface reflectance over the entire time period, enabling us to effectively detect changes that may be too slow or too subtle to measure over a shorter time span. A single map of natural regrowth and a single map of plantation were created for the entire study period, and these two maps included any tree growth event (whether natural or within a man-made plantation, respectively) regardless of permanence. We also used the same multi-epochal metric set to create a single map of stable natural land cover for the entire continent for our study period. The stable natural land cover class contained enormous spectral variation because it included many land cover types, such as forests (of varying cover and height), shrublands, wetlands, grasslands, and even bare ground. This class was predominantly characterized by its spectral stability throughout the 30+-year time period. The multi-epochal metric set enabled us to target this stability to discriminate between stable natural land cover and all other classes. As with the cropland maps, we used a bagged classification tree algorithm and manually labeled Landsat training data to create each of these three maps.

### Stratification and sample allocation

**Fig. 6:**
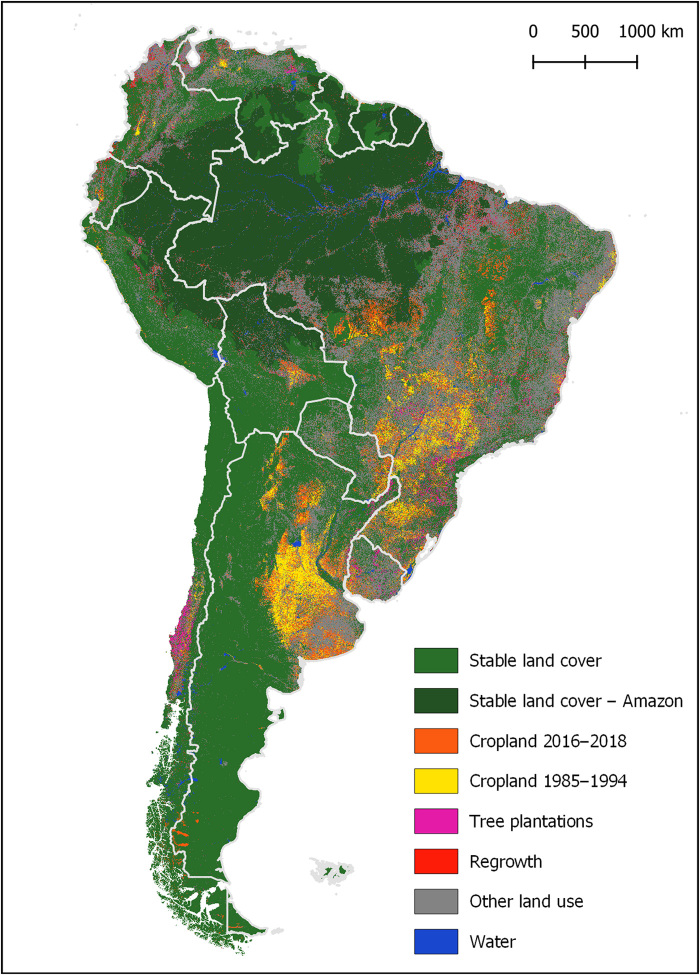
Study area and sampling strata.

We joined the aforementioned maps and an additional map of surface water (*58*) to create eight strata for sampling. The crop maps were aggregated into two strata: one maximum extent of all crop maps from 1985 to 1994 and another for all crop maps from 2016 to 2018. This approach was undertaken to target baseline crop areas and areas of crop gain through time. In addition, we created a separate stratum for natural land cover within the Amazon biome by overlapping our natural land cover map with the tropical rainforest ecozone (*59*). Once all layers were joined, the remaining area was assigned to a final stratum of “leftover” land, which corresponded largely to all land uses excluding cropland and plantations (predominantly pasture and settlements) (Fig. 6).

**Fig. 7:**
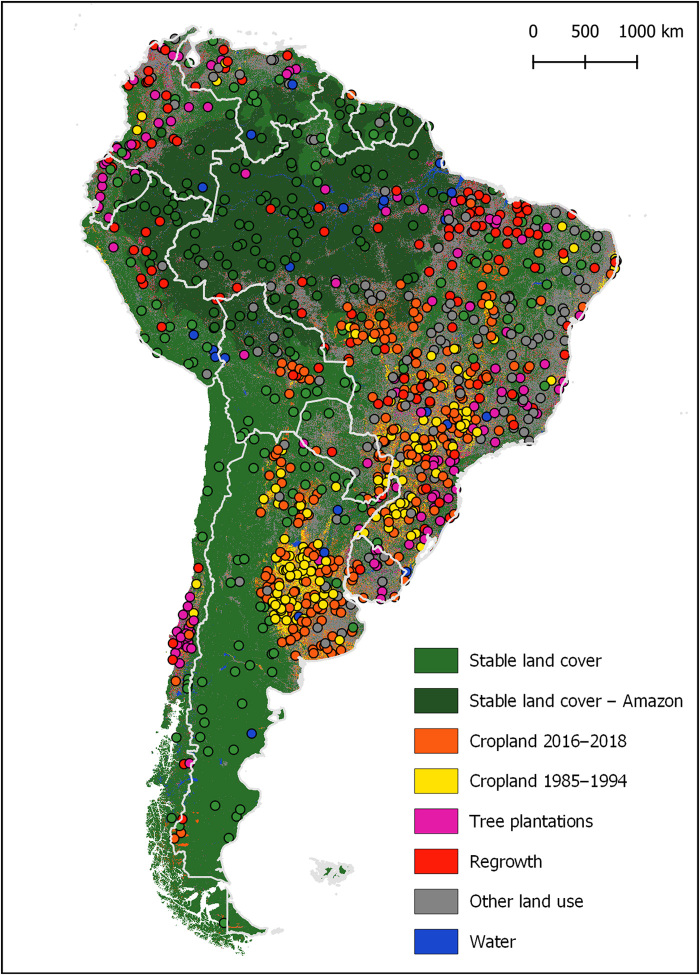
Sampled pixels by mapped strata.

A stratified random sample of 1000 units was drawn (Fig. 7), with each unit corresponding to a single Landsat pixel. The distribution of sample pixels can be found in table S1. Reference data for interpretation consisted of annual Landsat mosaics; bimonthly Landsat mosaics; time series of NDVI, SWIR 1, and red reflectance; and, where available, high-resolution Google Earth imagery. A user interface was created to aggregate all reference data and to collect interpretation data (see fig. S3). For each sampled pixel, we recorded the land use/land cover class for every year in the study period and every visible degradation event, as well as a confidence rating of our interpretation. For detailed information regarding the land cover/land use hierarchical legend used for interpretation and the class definitions, refer to Fig. 1 and table S2. Area estimates were derived from the sample using the R survey package (*60*) to implement the stratified sampling formulas following good practice recommendations (*16*–*18*, *61*, *62*). The sampling density allowed us to report area estimates for Brazil; Argentina; the combined area of Uruguay and Paraguay; and the combined area of Colombia, Venezuela, Peru, Bolivia, Ecuador, Suriname, French Guiana, and Guyana (Pan-Amazon, excluding Brazil). We were also able to derive area estimates for the following FAO ecological zones (*59*): tropical rainforest, tropical moist deciduous forest, tropical dry forest, and subtropical humid forest (fig. S4). We compared our area estimates to those derived from a large sample for Brazil obtained from the MapBiomas/LAPIG (*63*) land cover and land use change project and obtained comparable results (see Supplementary Text and fig. S5).

### Degradation

From our sample, we estimated the cumulative amount of land that was impacted by a degradation event since 1985. Depending on the ecosystem, degraded natural vegetation may fully recover and revert to its natural state after some time. Time series of Landsat imagery allowed us to detect certain degradation events (logging, fire, and edge effects), but assessing fine-scale impacts on structure or species composition was not feasible. Thus, we cannot rely on these data to assess whether a sampled pixel recovered from a degradation event. Although we refer to this land as degraded land, it should be interpreted as land that has been impacted by an observable degradation event at some point during the study period.

An important limitation of our method was that it likely omitted a portion of degradation events, leading to underestimation of the amount of land that was impacted by a degradation event since 1985. Omissions can happen if a degradation event occurred at a time that did not coincide with a satellite overpass or if a degradation event had impacts that were not detectable by the Landsat instrument. Both the fine spatial scale and ephemeral nature of degradation events limit their comprehensive quantification using Landsat data (*64*, *65*). We also do not account for land that was degraded before 1985. Degraded tree cover can typically only be detected by detecting the degradation event itself, so the amount of degraded tree cover at the beginning of the study period is underestimated. To compensate for this, we use the 1988 degradation area estimates for 1985 to 1987 for the purposes of conservatively estimating the total change in human impact over the study period. See the Supplementary Materials for a comparison of our results to those of a recent study (*64*).

### Human impact on natural land cover

To estimate human impact on natural land cover, we aggregated our land cover/land use classes into four categories based on the degree of modification or conversion and the intensity of land use. At the pinnacle of the human impact scale is the complete and permanent conversion of land from its natural state to intensive economic land uses (e.g., cropland, pasture, plantation, and infrastructure). In between intensive land use and intact natural land cover is a second category combining seminatural land and secondary forests. This second category includes land that has been subjected to human modification but is not discernably economically productive or intensive. Seminatural land consists of (i) transitional land, for example, poorly maintained or abandoned land uses such as pastures that feature natural vegetation recovery, and (ii) low-intensity, small-scale land uses that may be cyclical such as shifting cultivation within the Amazon forest. The remaining third and fourth categories both consist of natural tree cover that has experienced degradation or canopy and biomass loss due to human-induced disturbance. These last two categories are distinguished from each other by whether the initial degradation was caused by fire or caused by logging or other types of biomass removal.

Human impact, as we define it here, differs from the human footprint defined by Sanderson *et al.* (*13*) and others (*14*) in that they consider a continuum of human influence on the natural environment by including proxies such as population density (to incorporate the degree of impacts on land) and accessibility (to incorporate potentially impacted areas). It also differs from the “human appropriation of net primary production” (HANPP) indicator, which models how energy in the form of biomass is appropriated by human actions (*2*, *66*). Our results, on the other hand, focus only on measurable direct human impacts on the land at a 30-m spatial resolution and do not include distance or buffer measures nor vegetation modeling.

## SUPPLEMENTARY MATERIALS

Supplementary material for this article is available at http://advances.sciencemag.org/cgi/content/full/7/14/eabg1620/DC1
